# Quality of life up to 10 years after traumatic brain injury: a cross-sectional analysis

**DOI:** 10.1186/s12955-020-01391-3

**Published:** 2020-06-04

**Authors:** Katrin Rauen, Lara Reichelt, Philipp Probst, Barbara Schäpers, Friedemann Müller, Klaus Jahn, Nikolaus Plesnila

**Affiliations:** 1 Schoen Clinic Bad Aibling, Kolbermoorer Strasse 72, 83043 Bad Aibling, Germany; 2 Institute for Stroke and Dementia Research (ISD), University of Munich Medical Center, Feodor-Lynen-Straße 17, 81377 Munich, Germany; 3 Department of Geriatric Psychiatry, University Hospital of Psychiatry Zurich & Institute for Regenerative Medicine (IREM), University of Zurich, Minervastrasse 145, 8032 Zurich, Switzerland; 4 Institute for Medical Informatics, Biometry and Epidemiology (IBE), University of Munich, Munich, Germany; 5 German Center for Vertigo and Balance Disorders, University of Munich Medical Center, Munich, Germany; 6 Munich Cluster for Systems Neurology (Synergy), Munich, Germany

**Keywords:** Traumatic brain injury, Long-term outcome, Health-related quality of life after brain injury, QOLIBRI, Adaptation and resilience, Autonomy and cognition as decisive outcome factors for satisfaction, Depressive disorder, Anxiety, Psychiatric disorders after brain injury, TBI guidelines

## Abstract

**Background:**

Traumatic brain injury (TBI) is the leading cause of death and disability among children and young adults in industrialized countries, but strikingly little is known how patients cope with the long-term consequences of TBI. Thus, the aim of the current study was to elucidate health-related quality of life (HRQoL) and outcome predictors in chronic TBI adults.

**Methods:**

In this cross-sectional study, 439 former patients were invited to report HRQoL up to 10 years after mild, moderate or severe TBI using the QOLIBRI (Quality of Life after Brain Injury) questionnaire. The QOLIBRI total score has a maximum score of 100. A score below 60 indicates an unfavorable outcome with an increased risk of an affective and/or anxiety disorder. Results were correlated with demographics and basic characteristics received from medical records (TBI severity, etiology, age at TBI, age at survey, time elapsed since TBI, and sex) using regression models. Differences were considered significant at *p* <  0.05.

**Results:**

From the 439 invited patients, 135 out of 150 in principle eligible patients (90%) completed the questionnaire; 76% were male, and most patients experienced severe TBI due to a traffic-related accident (49%) or a fall (44%). The mean QOLIBRI total score was 65.5 (± 22.6), indicating good HRQoL. Factors for higher level of satisfaction (*p* = 0.03; adjusted R^2^ = 0.1) were autonomy in daily life (*p* = 0.03; adjusted R^2^ = 0.09) and cognition (*p* = 0.05; adjusted R^2^ = 0.05). HRQoL was weakly correlated with initial TBI severity (*p* = 0.04; adjusted R^2^ = 0.02). 36% of patients reported unfavorable HRQoL with increased risk of one (20%) or two (16%) psychiatric disorders.

**Conclusions:**

The majority of chronic TBI patients reported good HRQoL and the initial TBI severity is a slight contributor but not a strong predictor of HRQoL. Autonomy and cognition are decisive factors for satisfied outcome and should be clearly addressed in neurorehabilitation. One third of patients, however, suffer from unsatisfactory outcome with psychiatric sequelae. Thus, an early neuropsychiatric assessment after TBI is necessary and need to be installed in future TBI guidelines.

## Introduction

Traumatic brain injury (TBI) is the most common cause of death and disability among children and young adults in industrialized countries and has become increasingly prevalent among the elderly [1]. Worldwide, the annual incidence of TBI varies and ranges up to 500/100,000 [2–4]. Thus, TBI is even more frequent than stroke with estimates of an annual incidence of 258/100,000 [5]. Further, TBI resulted in the loss of 247.6 Mill. disability-adjusted life years (DALY) between 1990 and 2013 compared to stroke with only 102 Mill. DALY lost in 2010 [5–7]. The enormous health and socio-economic burdens affect primarily young working populations and its heterogeneous and often underdiagnosed psychiatric sequelae hamper patient’s reintegration in their premorbid working environment.

To date, TBI research has primarily focused on the acute pathophysiology underlying the primary and secondary brain injury (i.e., life-threatening brain edema and increased intracranial pressure). Despite numerous positive results obtained with basic TBI research, no treatment has yet been successfully translated from bench to bedside [8]. Thus, interest has also shifted to studying long-term sequelae among TBI survivors, who often experience a variety of serious chronic neurological and/or psychiatric conditions [9–14].

In addition to physical and cognitive disabilities, TBI patients often suffer from psychiatric disorders—namely affective, anxiety, posttraumatic stress disorders and/or sleep disturbances [11, 13, 15, 16]. However, even though TBI survivors and their caregivers may benefit from early psychiatric diagnostics and supportive neuropsychiatric treatment—facilitating adaptation and resilience after the brain impact—these heterogeneous symptoms are not routinely assessed during neurorehabilitation, and thus have neither been the focus during early neurorehabilitation of TBI patients nor have been implemented in TBI guidelines, so far [13, 14, 17–19].

Previous TBI outcome studies have primarily assessed patients’ physical status using the extended Glasgow Outcome Scale within 6 months after TBI, as about 85% of recovery occurs within this time frame [20]. But, today it is well-known that patients recover beyond 6 months after their head impact and outcome is not only related to patient’s mental and physical status, but also to their self-assessed health-related quality of life (HRQoL) [21–25]. Furthermore, approximately one third of chronic TBI (cTBI) patients present with ongoing functional and/or cognitive decline, suggesting a progressive pathophysiology, when evaluated up to 10 years after their head impact [26, 27]. Thus, the raising question is how patients cope with the long-term consequences after TBI based on their self-assessment and indicating the patient’s well-being over time.

The Quality of Life after Brain Injury (QOLIBRI) is a health-related and disease-specific instrument for assessing quality of Life (QoL) after brain injury. It has been internationally validated in a variety of languages, including German [28, 29]. The QOLIBRI total score ranges from 0 to 100, representing the lowest and highest HRQoL, respectively, and has good internal consistency (alpha = 0.95) and good test-retest reliability (ICC = 0.91) [28, 30–32]. The QOLIBRI was systematically related to the patient’s emotional state, functional outcome, comorbidity, and generic health, which were assessed by using the Hospital Anxiety and Depression Scale, the Glasgow Outcome Scale, a health questionnaire regarding 28 comorbidities, and the Short-form (36) health survey questionnaire, i.e. the SF-36, which is a generic instrument for measuring HRQoL; thus, the QOLIBRI is well-suited for use in measuring self-reported outcome following TBI and can be completed within 7 to 10 min. In contrast, other well-established instruments for measuring generic quality of life—including the World Health Organization Quality of Life instruments, i.e. the WHOQOL-100, WHOQOL-BREF, as well as the SF-36, SF-12, and SF-8 questionnaires—are more time-consuming and/or cover fewer TBI-related sequelae. A recent meta-analysis revealed that the SF-36 is still the most commonly used tool for assessing HRQoL in TBI patients [33]. Despite its wide use in TBI research, however, the SF-36 has several disadvantages compared to the QOLIBRI instrument when used in patients with chronic TBI (cTBI). First, the SF-36 does not adequately cover the most important domains of cognition and social relationship in cTBI patients. Second, patients with cTBI are often unable to complete the SF-36 by themselves due to TBI-related cognitive and/or motor deficits; in contrast, self-completion is not a prerequisite of the QOLIBRI instrument—playing a role in 28% of our patients. Finally, the SF-36 does not cover TBI-specific factors with respect to the most common frontotemporal damages through the brain impact, that might result in changes of the patient’s mental health presented as depression, anxiety disorder and/or cognitive dysfunction [33]. Thus, these disadvantages underline that the SF-36 is not ideally suited for assessing patients with cTBI. A meta-analysis on HRQoL after TBI found that 2 out of 9 validation studies—with 573 and 153 patients—used the QOLIBRI instrument to analyze long-term HRQoL up to 15 years post-injury [33], thereby reflecting the relative paucity of evidence in the field of cTBI.

Taking together, little is known how patients cope with the neuropsychiatric long-term consequences and predicting outcome factors for satisfactory outcome are still insufficiently clarified following TBI [34, 35]. Hence, the aim of this study was to evaluate long-term HRQoL including the risk of psychiatric sequelae as well as to elucidate potential predictors for good long-term outcome in post-TBI adults.

## Methods

### Study design and procedure

**Fig. 1:**
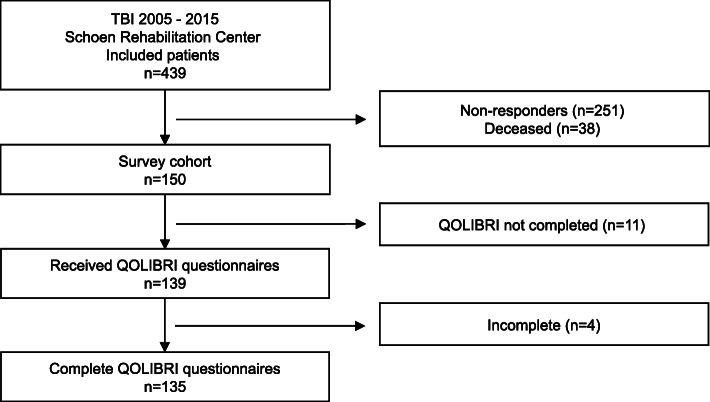
Flow-chart depicting the survey cohort of patients with chronic TBI. In this cross-sectional study, 135 out of the 150 survey participants—representing a net response rate of 90%—reported on their health-related quality of life (HRQoL) up to 10 years following neurorehabilitation due to traumatic brain injury using the QOLIBRI (Quality of Life after Brain Injury) questionnaire

For this cross-sectional study, 439 consecutively admitted TBI patients were identified in the electronic database of the Schoen Rehabilitation Center, Bad Aibling, Germany, who underwent neurorehabilitation due to a mild, moderate or severe TBI between January 1, 2005 through June 30, 2015. Before admission to the Schoen Rehabilitation Center, these patients received critical care in a hospital of the Southern Upper-Bavaria Trauma Network, thereby assuring a comparable and high standard of critical and surgical care. To gain knowledge on how patients cope with the long-term consequences after TBI, these patients were invited in writing to participate in this cross-sectional analysis on November 4, 2015 (Fig. 1 and supplementary Fig. S1). The letter contained a cover letter with a request to answer the QOLIBRI questionnaire, the QOLIBRI questionnaire as well as a prepaid return envelope. Thus, HRQoL was assessed up to 10 years following TBI using the health-related and disease-specific QOLIBRI questionnaire. According to local legislation and the ethics committee of the Ludwig-Maximilians University, Munich, Germany, ethical approval was not required for this study as this cross-sectional analysis was performed as catamnesis and all data from medical records were anonymously used. In detail, this catamnesis was performed to gain knowledge how former patients cope with the long-term consequences after neurorehabilitation due to a mild, moderate or severe TBI, which does not require an ethical approval. In accordance to this approach, a second contact of patients was not allowed and not performed.

### Participants and eligibility criteria

Patients equal or older than 18 years at survey who experienced an open or closed, mild, moderate, or severe TBI were eligible; patients with either intracranial bleeding without a TBI or a chronic subdural hematoma were excluded.

### QOLIBRI questionnaire

The QOLIBRI is a form-based questionnaire designed to measure HRQoL in patients following brain injury. It has been internationally validated in a variety of languages, including German [28, 29]. The QOLIBRI total score ranges from 0 to 100, representing the lowest and highest HRQoL, respectively. It comprises the following six scales with 37 items: cognition (7 items), self (7 items), daily life & autonomy (7 items), social relationships (6 items), emotions (5 items), and physical problems (5 items). Two major key aspects in life—satisfaction and bothered items, i.e. restrictions—are assessed by merging items 1 through 4 (cognition, self, daily life & autonomy, and social relationships) and items 5 and 6 (emotions and physical problems) [31], with an additive maximum score of 400 (merged items 1–4 with each having a maximum score of 100) and 200 (merged items 5–6 with each having a maximum score of 100), respectively. A QOLIBRI total score of 60 or higher represents good HRQoL; a score below 60 indicates unsatisfactory HRQoL with an increased risk of having either an affective or anxiety disorder, and a score below 40 indicates an increased risk of having both disorders [29]. In the case of severe cognitive and/or motor impairment, the patient’s caregiver helped the patient complete the questionnaire. HRQoL was then quantified and the results were correlated with the following demographic and basic characteristics, namely *i)* TBI severity (initial GCS), *ii)* TBI etiology (traffic accident/fall), *iii)* the patient’s age at the time of TBI, *iv)* the patient’s age at the time of the survey, *v)* the time elapsed between TBI and completion of the questionnaire, and *vi)* the patient’s sex (male/female).

### Demographic data and basic characteristics

To rule out non-responder bias, the following data are given for the main unit (*n* = 439), the non-responders (*n* = 251) and the QOLIBRI cohort (*n* = 135) and were statistically compared between the non-responders and the QOLIBRI cohort: *i*) TBI severity, *ii*) TBI etiology, *iii*) age at the time of TBI, *iv*) age at the time of the survey, *v*) time elapsed since TBI, *vi*) sex distribution of the patient cohort, *vii*) whether decompressive craniectomy, *viii*) ICP monitoring/ a shunt device, and *ix*) a tracheostomy was performed as well as *x)* time to onset of neurorehabilitation, *xi)* duration of neurorehabilitation, *xii)* functional status at admission to, and *xiii)* at discharge from neurorehabilitation. The relative frequency (%) of each TBI severity level was quantified using the categories: mild (TBI I°: GCS 13–15), moderate (TBI II°: GCS 9–12), or severe (TBI III°: GCS 3–8) injury, based on the initially documented score on the Glasgow Coma Scale (GCS) [36], which was extracted from the medical referral letter that was sent to the Schoen Rehabilitation Center. The relative frequency (%) of each TBI etiology was determined for the following three categories: traffic accidents, falls, and other.

### TBI severity and missing data

In this analysis of chronic TBI patients, we addressed the common lack of GCS documentation regarding TBI severity as followed [25, 37]. 62% of patients (*n* = 84) were initially classified for their TBI severity (GCS) and were included in our correlation analysis (model 1 and 2). For the descriptive analysis of demographic data, we included all 135 patients. For the overall data interpretation, we assumed that most probably 58 to 70% underwent either a primary severe TBI or a secondary deterioration resulting in severe secondary brain injury, indicated by the portion of decompressive craniectomy and ICP monitoring during the acute treatment (Table 1).

**Table 1:** Demographic and basic characteristics did not differ between the non-responders (n=251) and the QOLIBRI cohort (n=135) regarding most of the analyzed parameters. However, patients of the QOLIBRI cohort gained better function at discharge from neurorehabilitation. TBI severity: mild (GCS 13-15), moderate (GCS 9-12), severe (GCS 3-8); mobile functional status represents a modified Rankin Scale of 0 to 3; immobile functional status represents a modified Rankin Scale of 4 to 5

	Main unit *n* = 439	Non-responder *n* = 251	QOLIBRI cohort *n* = 135	*p*-Value
**TBI severity** mild/ moderate/ severe/ n.s. (%) ^b^	9.8/ 8.4/ 32.1/ 49.7	8.0/ 6.4/ 33.5/ 52.1	13.3/ 13.3/ 35.6/ 37.8	0.08
**TBI etiology** traffic accidents/ falls/ others (%) ^a^	45.1/ 45.3/ 9.6	43.4/ 47.0/ 9.6	48.8/43.9/ 7.3	0.22
**Age at TBI** (mean ± SEM) ^b^	53.4 ± 2.4	51.03 ± 1.32	47.5 ± 1.7	0.08
**Age at survey** (mean ± SEM) ^b^	56.7 ± 1.0	56.2 ± 1.3	53.1 ± 1.6	0.13
**Time since TBI** (mean ± SEM) ^b^	5.4 ± 0.1	5.2 ± 0.2	5.7 ± 0.3	0.14
**Sex distribution** male/ female (%) ^a^	74/ 26	73/ 27	76/ 24	0.63
**Decompressive craniectomy** (%) ^a^	30	32	27	0.42
**ICP monitoring or permanent shunt device** (%) ^a^	60	62	57	0.38
**Tracheostomy** (%) ^a^	50	52	44	0.23
**Time to onset of neurorehabilitation**^b^ (days, mean ± SEM)	29 ± 1.6	29.8 ± 2.5	26.5 ± 2.3	0.76
**Duration of neurorehabilitation**^b^ (days, mean ± SEM)	49.7 ± 2.3	51.8 ± 3.3	38.8 ± 2.9	0.08
**Functional status at admission** (mobile (mRS 0–3)/ immobile (mRS 4–5)/ n.c. (%)) ^a^ (mean ± SEM) ^b^	8.7/ 88.8/ 2.5 4.53 ± 0.04	10.8/ 89.2/ 0 4.53 ± 0.06	5.2/ 94.1/ 0.7 4.6 ± 0.07	0.09 0.85
**Functional status at discharge** (mobile (mRS 0–3)/ immobile (mRS 4–5)/ n.c. (%)) ^a^ (mean ± SEM) ^b^	61.5/ 36/ 2.5 2.95 ± 0.07	58.6/ 41/ 0.4 3.13 ± 0.1	80.7/ 18.5/ 0.8 2.25 ± 0.1	< 0.001* < 0.001*

*n.c.* not classified in the medical record, *n.s*. not specified in the medical record, TBI: traumatic brain injury, ICP: intracranial pressure, mRS: modified Rankin Scale

^a^ Fisher test for categorical variables

^b^ Mann-Whitney-U test for numeric variables

* statistical difference with *p* <0.05

### Data management

Each QOLIBRI questionnaire returned was checked for completeness, and a self- or caregiver-assessed rating was recorded. Each QOLIBRI responder was then assigned an interim ID number. The QOLIBRI scores were added to the 13 demographic results listed above, which were obtained from the medical records using the interim ID numbers. Finally, all ID numbers were removed, and the entire data set was anonymized.

### Statistical analysis

Statistical analysis was performed using R (R Core Team, Vienna, Austria). Group differences were calculated between the QOLIBRI cohort (*n* = 135) and the non-responders (*n* = 251) using the Mann-Whitney-U test for numeric or the Fisher test for categorical variables. All QOLIBRI data were tested for a Gaussian distribution using a QQ plot. A multivariate regression model (model 1) was used to assess the parameters that influence HRQoL measured using the QOLIBRI total score. HRQoL was modeled as a dependent variable and as a function of the following potential predictors (as independent variables): *i*) TBI severity (GCS), *ii*) TBI etiology, *iii*) age at the time of TBI (calculated by subtracting the age at the time of the survey from the time elapsed since TBI), *iv*) age at the time of the survey, *v*) time elapsed since TBI, and *vi*) sex distribution. A second multivariate regression model (model 2) was used to analyze the putative effect of TBI severity (GCS) on the QOLIBRI total score, on the two major aspects in life, namely satisfaction and restrictions, and on each of the six scales. Patients with missing data of TBI severity (*n* = 51) were excluded for regression analysis (model 1 and 2), thus these regression models included 84 out of 135 patients. Data are reported as a relative frequency (%), the mean ± SEM (demographics), the mean ± SD (QOLIBRI results), or the median with interquartile range (IQR_25–75_). Differences were considered significant at *p* <  0.05. Effect size was assessed using the determination coefficients R^2^ and adjusted R^2^. Four of the original 139 returned questionnaires were incomplete and were therefore excluded from the analysis.

## Results

### Response rate

One hundred thirty-five out of 439 consecutive admitted TBI patients of the electronic database of the Schoen Rehabilitation Center, completed the analysis, from now on termed as the “QOLIBRI cohort” (Fig. 1). In detail, 251 patients did not respond to our written invitation to participate and at least 38 out of 439 potentially eligible cTBI patients had already deceased, as indicated by their relative’s answer. One hundred fifty out of 401 (37%) former patients responded to our inquiry. Eleven of the responding patients did not return the QOLIBRI questionnaire, and an additional 4 responders did not complete the questionnaire. Thus, 135 out of 150 (90%) chronic cTBI (cTBI) patients of our survey cohort provided complete information regarding their HRQoL measured using the QOLIBRI questionnaire (Fig. 1).

### Demographic data and basic characteristics

The QOLIBRI cohort (*n* = 135) and the non-responders (*n* = 251) did not differ with respect to TBI severity (*p* = 0.08), TBI etiology (*p* = 0.22), age at TBI (*p* = 0.08), age at survey (*p* = 0.13), elapsed time since TBI (*p* = 0.14), sex distribution (*p* = 0.63), decompressive craniectomy (*p* = 0.42), ICP monitoring/ shunt device (*p* = 0.38), tracheostomy (*p* = 0.23), time to onset of neurorehabilitation (*p* = 0.76), duration of neurorehabilitation (*p* = 0.08), and functional status at admission (*p* = 0.09). However, patients of the QOLIBRI cohort gained better functional status at discharge from neurorehabilitation with a mean (± SEM) modified Rankin Scale (mRS) of 2.3 ± 0.1 in comparison to the non-responders (n = 251) with a mean (± SEM) mRS of 3.1 ± 0.1 (*p* < 0.001). Thus, patients of the QOLIBRI cohort gained on average good mobility and independence in activities of daily living though unable to carry out all previous activities compared to the non-responders, who remained on average moderate disabled though able to walk unassisted (Table 1). Among the 135 patients in the QOLIBRI cohort, 13, 13, and 36% had mild, moderate, or severe TBI, respectively; TBI severity was not classified for the remaining 38% of patients. With respect to TBI etiology, 49% of cases were due to a traffic accident, 44% were due to a fall, and the remaining 7% were due to another cause. The mean (± SEM) age at the time of the survey was 53.1 ± 1.6 years, and 76% of the participants were male. 27% of patients underwent decompressive craniectomy in the acute phase, representing a life-threatening increased intracranial pressure (ICP) or the need for a neuroprotective intervention. 57% of patients required either ICP monitoring during the acute treatment—using external ventricular drain or intraparenchymal probe—or a permanent ventriculo-peritoneal shunt system, all interventions indicating either primary or secondary severe brain damage.

### TBI severity and health-related quality of life

**Fig. 2:**
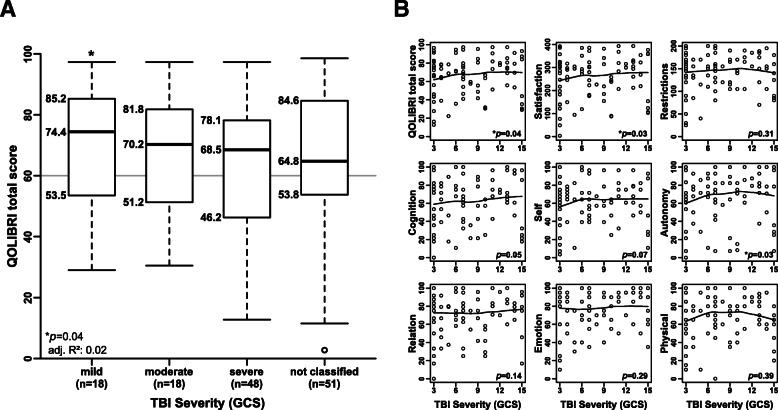
TBI severity is not a strong predictor of health-related quality of life. **a** TBI severity was weakly correlated with the QOLIBRI total score (*p* = 0.04; adjusted R^2^ = 0.02). **b** The QOLIBRI questionnaire can be used to distinguish two major key aspects in life, satisfaction (obtained by merging the scales cognition, self, daily life & autonomy, and social relationships) and restrictions (obtained by merging the scales emotions and physical problems). TBI was weakly correlated with satisfaction (*p* = 0.03, adjusted R^2^ = 0.1), daily life & autonomy (*p* = 0.03; adjusted R^2^ = 0.09), and cognition (*p* = 0.05; adjusted R^2^ = 0.05). In contrast, TBI severity was not correlated with restrictions (*p* = 0.31; adjusted R^2^ = 0.08). TBI severity was poorly documented in the medical records of 51 patients (38%); thus, TBI severity was not classified in these 51 patients. GCS: Glasgow Coma Scale

Initial TBI severity was weakly correlated with HRQoL (*p* = 0.04; adjusted R^2^ = 0.02) (Fig. 2). The median QOLIBRI total score was 74.4 (IQR_25–75_: 53.5–85.2), 70.2 (IQR_25–75_: 51.2–81.8), and 68.5 (IQR_25–75_: 46.2–78.1) in the patients with mild, moderate, and severe TBI, respectively. In contrast, HRQoL was not correlated with TBI etiology, age at TBI, age at survey, time elapsed since TBI, or sex distribution (Supplementary Table S1 and Fig. S2). The model’s effect size was low, with an R^2^ value of 0.09 and an adjusted R^2^ of 0.02, indicating a weak correlation, revealing that each 1-point increase in the GCS is associated with a 1.59-point increase in the QOLIBRI total score.

Our analysis also revealed that TBI severity influenced the patient’s level of satisfaction (*p* = 0.03; adjusted R^2^ = 0.1), one of the QOLIBRI key aspects influenced primarily by the factors of daily life & autonomy (*p* = 0.03; adjusted R^2^ = 0.09) and cognition (*p* = 0.049; adjusted R^2^ = 0.05) (Figs. 2b, Supplementary Table S2). In contrast, TBI severity had no effect on the second QOLIBRI key aspect, restrictions (*p* = 0.31; adjusted R^2^ = 0.08), which was obtained by merging the scales emotions (*p* = 0.29; adjusted R^2^ = 0.04) and physical problems (*p* = 0.39; adjusted R^2^ = 0.04).

**Fig. 3:**
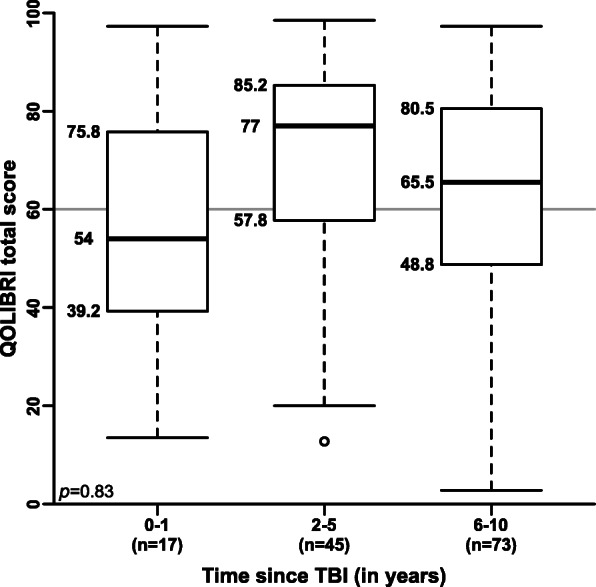
Time effects on health-related quality of life following TBI. The first year following TBI seems to be crucial for patient’s rehabilitation and adaption, as 13% of patients (*n* = 17) reported having insufficient HRQoL with a median QOLIBRI total score of 54. Thus, psychiatric evaluation and support should be provided as early as possible after traumatic brain injury

### Health-related quality of life during the first year following TBI

Seventeen patients (13%) of the QOLIBRI cohort were included during the first year after their TBI. These 13% of patients (*n* = 17) reported insufficient HRQoL with a median QOLIBRI total score of 54 (IQR_25–75_: 39.2–75.8) (Fig. 3). Two to 5 years after TBI, 33% of patients (*n* = 45) reported good HRQoL with a median QOLIBRI total score of 77 (IQR_25–75_: 57.8–85.2). Finally, 6 to 10 years after TBI, 54% of patients (*n* = 73) reported good HRQoL with a median QOLIBRI total score of 65.5 (IQR_25–75_: 48.8–80.5) (Fig. 3).

### Health-related quality of life and sex distribution after TBI

Among the 135 participants, 76% (*n* = 102) were male and 24% (*n* = 33) were female. Sex was not correlated with HRQoL following TBI (*p* = 0.91; adjusted R^2^ = 0.02). The median QOLIBRI total scores among the male and female patients were 69.9 (IQR_25–75_: 53.5–84.0) and 61 (IQR_25–75_: 48.8–75.0), respectively (Supplementary Fig. S2 D).

### Health-related quality of life and increased risk of psychiatric sequelae after TBI

**Fig. 4:**
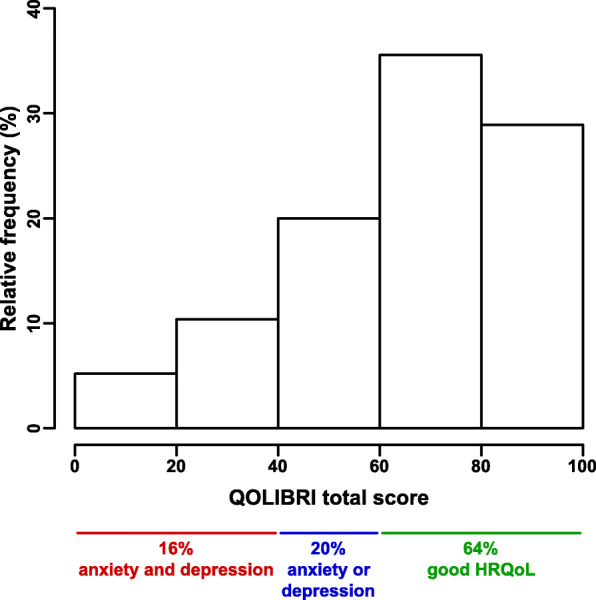
Health-related quality of life and risk of psychiatric sequels after TBI. Health-related quality of life was assessed in 135 patients with mild, moderate, or severe TBI up to 10 years after neurorehabilitation. The QOLIBRI total score ranges from 0 to 100, representing the lowest and highest quality of life, respectively. Based on the QOLIBRI total scores, 64% of chronic TBI patients indicated good quality of life, 20% of patients had either an increased risk of anxiety or depressive disorder, and 16% of patients an increased risk of both psychiatric disorders

Among our cohort of cTBI patients, 64% indicated good HRQoL, reflected by a mean (± SD) QOLIBRI total score of 65.5 ± 22.6, a value well in line with the general population and the QOLIBRI validation cohort (Supplementary Table S3) [38]. The remaining 36% of patients had a QOLIBRI total score below 60, indicating an increased risk of a depressive and/or anxiety disorder. In detail, 20% of patients had either an increased risk of a depressive or anxiety disorder, and 16% of patients for both disorders (Fig. 4). Furthermore, the majority of patients reported on average good HRQoL in all six QOLIBRI scales with the following mean (± SD) scores: cognition (62.4 ± 27.2), self (61.1 ± 25.6), daily life & autonomy (63.5 ± 31.0), relationships (69.3 ± 23.7), emotions (75.0 ± 23.9), and physical problems (66.1 ± 24.1), values again well in line with the previously published QOLIBRI validation cohort of 795 chronic TBI patients [38], indicating that the results of the current study are robust and in line with already published results (Supplementary Table S3 and Fig. S3).

## Discussion

In this cross-sectional study, 135 chronic TBI patients who were representative in most of the analyzed demographics and basic characteristics of the entire cohort of 439 adult, chronic TBI patients reported their health-related and disease-specific quality of life (HRQoL) up to 10 years after experiencing mild, moderate, or severe TBI. Approximately two thirds of the 135 patients reported good HRQoL. The initially classified TBI severity was only a slight contributor to—but not a strong predictor of—HRQoL among our chronic TBI patients. In contrast, none of the other parameters including TBI etiology, age at the time of TBI, age at the time of the survey, time elapsed since TBI, or sex distribution was correlated with HRQoL. One third of our patients reported unfavorable HRQoL with limited autonomy and cognition as decisive factors for unsatisfactory outcome—associated with an increased risk of anxiety and/or depressive disorders. Furthermore, the first year following TBI revealed unfavorable HRQoL with an increased risk of psychiatric sequelae, suggesting that early neuropsychiatric treatment is crucial to support patient’s adaption and resilience. The presented results are in line with the QOLIBRI validation study having analyzed 795 TBI patients (Supplementary Table S3) as well as with a Dutch study, which reported good HRQoL in 62% of patients having experienced diffuse axonal injury (DAI) [21, 38]. However, there are still controversial findings in the literature regarding age and sex differences that need further attention—most probably explainable due to the heterogeneity of TBI cohorts.

### A representative TBI cohort

The response and participation rate were 37% (150 of 401 alive patients) and 90% (135 out of 150 participant of our survey cohort), respectively. These values are relatively high, given (1) the long-follow up period of up to 10 years after TBI, (2) the high age of participating patients, (3) the severity of TBI, and (4) the fact that a second contact by telephone was not permitted by German data protection laws. This response rate is consistent with a large German multicenter epidemiological survey of 4307 pediatric and adult TBI patients evaluated 1 year after TBI, which yielded a primary response rate of 40% [37].

Most demographic data and basic characteristics did not differ between the QOLIBRI cohort and the non-responders. However, patients of the QOLIBRI cohort gained better functional status at discharge from neurorehabilitation with a mean modified Rankin Scale of 2, indicating slight disability with good mobility and independence in activities of daily living though unable to carry out all previous activities, while the non-responders remained moderate disabled—indicating that a non-responder bias cannot fully be excluded, but most parameters were comparable to the entire cohort of 439 TBI patients, and thus the QOLIBRI cohort is most likely representative for this larger group of chronic TBI patients.

All participants underwent neurorehabilitation at Schoen Rehabilitation Center in Bad Aibling, Germany—one of the largest neurorehabilitation units in Europe—and were primarily referred by certified TBI centers within the Southern Upper-Bavaria Trauma Network, thereby providing the highest standard of medical care to patients in the acute phase of TBI. Despite the common lack of GCS documentation in 38% of medical records [25, 37], it is reasonable to assume that most patients experienced a severe brain injury, namely 36% due to the initially documented GCS and up to 34% due to secondary brain injury. Specifically, 27% of patients underwent decompressive craniectomy and 57% received either intracranial pressure (ICP) monitoring to detect and guide therapy of intracranial hypertension and brain edema or a permanent ventriculo-peritoneal shunt system, procedures indicating severe brain damage. Based on a close examination of the relative proportions of patients who underwent decompressive craniectomy and/or received ICP monitoring and/or a shunt device, 58 to 70% were either primarily or secondarily severe brain injured, thus our cohort is comparable to the QOLIBRI validation cohort [38]. Other factors regarding our cohort, including TBI etiology and sex distribution, were consistent with the literature [23, 28, 39].

On average, our cohort was 20 years older than the cohort used to validate the QOLIBRI questionnaire, but this age difference did not change HRQoL and supports our finding that age is not a contributor to HRQoL in cTBI patients [38]. In contrast, the Dutch study found younger age as an independent predictor of lower HRQoL after DAI, while others report older age as an independent risk factor for a decreased HRQoL or likewise no age effects [21, 40, 41]. In detail, Scholten et al. analyzed HRQoL using the SF-36 instrument at 6 and 12 months after predominantly mild and after moderate to severe TBI with a mean age of 44 years (range 27–57) compared to 53 (range 18–85) years at survey in our QOLIBRI cohort [40]. This study emphasizes—well in line with our findings—that HRQoL increases over time until 12 months after TBI and this finding was most evident in the primarily mildly injured patients. Furthermore, severer injuries, less functional recovery, older age and female sex negatively correlated with better HRQoL but were potentially influenced by the 56 and 84% of patients lost to follow up at 6 and 12 months, respectively. Besides the initial TBI severity, i.e. patients of our QOLIBRI cohort reported better HRQoL after milder brain injuries—those findings are contradictory to the QOLIBRI cohort. Van Eijck and colleagues analyzed 86 chronic TBI patients aged 16–87 years on average up to 57 months (range 14–100 months post-TBI) after DAI due to a TBI with younger patients reporting less good HRQoL. This age-related finding which contrasts our current results, might be explained by the cognitive impairment after DAI [21]. Nevertheless, the QOLIBRI validation cohort included 795 chronic TBI patients—aged 17 to 68 years—3 months and up to 18 years after TBI, thus highly comparable with our QOLIBRI cohort, with only very weak correlations (r ≤ 0.11) for age effects, education, time since injury, and severity of injury (GCS) with HRQoL [30]. However, evidence of HRQoL in the elderly after TBI is scarce and might rather be associated with the preinjury and psychosocial capabilities than with the injury-related factors [41]. Taking together, age might be relevant for HRQoL in a variety of subgroups including TBI injury patterns, timepoint of follow up and potentially sample sizes and certainly need further attention in future long-term HRQoL outcome studies.

### Quality of life in the general German population

During the first year after TBI, HRQoL was reduced in our cohort indicating an increased risk of psychiatric sequelae and maladaptation to the post-TBI changes and this relevant finding is in line with the literature [24, 33, 40, 42]. Beyond one year post-TBI, HRQoL was even slightly better in our cohort than in the general German population and comparable to previous findings [22, 37, 43]. We suggest that future studies should investigate if good individual adaptation and resilience to the TBI-related changes are associated with better HRQoL outcome and the absence of psychiatric sequelae. Thus, these two factors—adaptation and resilience—should be prospectively focused and might be contributors to good HRQoL and long-term outcome after TBI.

### Psychiatric sequelae after brain injury

Psychiatric disorders such as anxiety and depression are common in the general population with a 12-months prevalence of 18 and 9.5%, respectively [44]. Following TBI, psychiatric sequelae are relevant and frequencies vary in the literature ranging from 18 to 83% due to methodological issues such as diagnostic criteria, injury severity and elapsed time since the brain impact [45]. Especially, anxiety and affective disorders are most relevant after TBI with data ranges up to 70% for anxiety and between 25 and 77% for depressive disorders [9, 10, 46]. Approximately 40% of TBI patients even suffer from more than two psychiatric sequelae [10, 46].

In our chronic TBI cohort, one third of patients suffered from insufficient HRQoL associated with an increased risk of psychiatric sequelae, namely anxiety and/or depressive disorders. This finding is in line with results of a prospective study analyzing 817 TBI patients of which a total of 31% reported psychiatric disorders at 12 months after TBI [13]. The latter finding suggests an increased risk for mental health changes such as depressive and/or anxiety disorders after TBI with the need for routine diagnostic of these posttraumatic sequelae that hamper patient’s quality of life. Furthermore, depressive symptoms might increase over time and psychiatric sequelae are a major reason for rehospitalization after TBI [22, 47]. Whether these psychiatric sequelae are associated with the functional decline described in one third of chronic TBI patients is still not elucidated. However, the link between the posttraumatic heterogeneous psychiatric sequelae and patient’s outcome becomes more and more obvious and need our attention throughout neurorehabilitation.

### Study limitations

This study has several limitations that warrant discussion. First, the cross-sectional study design with the sample size of 135—albeit representative for the entire cohort of 439—chronic TBI patients allows descriptive conclusions. But, a recent systematic review and meta-analysis on post-TBI HRQoL revealed that the majority of the included 49 studies between 1991 to 2013 analyzed less than 100 post-TBI patients [33]. Two further TBI studies analyzed HRQoL of 60 and 51 patients 10 years after TBI, respectively [22, 25]. Thus, the sample size of 135 chronic TBI patients in our study seems to be appropriate to highlight the need for psychiatric assessment on a regular base after TBI, especially when regarding the age range up to the 85-years-old, the long time period of up to 10 years after TBI as well as the given evidence, so far. Second, premorbid psychiatric sequelae, comorbidities, education, employment, living environment, injury patterns, and pharmacotherapy were not available. Third, neuroimaging data were not included to further characterize injury patterns as *i)* patients were referred from different trauma centers to neurorehabilitation, i.e. neuroimaging was per se less comparable or not available, *ii)* TBI patients—except for clinical deterioration or scheduling the bone flap replacement following decompressive craniectomy—do not get a routine neuroimaging while in neurorehabilitation, *iii)* state-of-the-art imaging for DAI (diffusion tensor imaging (DTI), tractography and susceptibility weighted imaging using a gradient recall echo (GRE-SWI)) was not available at the time when most of our patients were injured, i.e. more than 10 years ago, and *iv)* a multilevel diagnostic approach including neuroimaging and fluid biomarkers is recommended, but has not been implemented in the clinical routine, and thus were not available for our cohort [48]. But, DAI does not seem to influence HRQoL up to 5 years after TBI, therefore the injury pattern itself might be a less relevant factor for long-term outcome [49]. Fourth, functional status and comorbidities were not assessed in this survey-like cross-sectional study as written self-rating of functional status is most probably a less valid approach to get sustainable data. Fifth, caregiver’s quality of life and external assessments to elucidate a more objective perspective of the patients’ outcome was not done, but seem less relevant as indicated in the literature so far [50, 51]. Sixth, although a total score below 60 on the QOLIBRI questionnaire indicates an increased risk of psychiatric sequelae, a precise cut-off score has not been established; accordingly, our results on psychiatric disorders must be interpreted with care, but might help to implement cut-off scores and highlight the need for further evidence. Seventh, the initial GCS was not documented in 38% of cases in our cohort—a well-known finding in previous studies [25, 37]. Finally, the results’ generalizability might be limited due to the following issues: *i)* the age span of 18- to 85-year-olds, *ii)* the German population, *iii)* the heterogeneity of TBI in our cohort, and *iv)* treatment regimens including neurorehabilitation [21, 22]. Nevertheless, age does not seem to be relevant for long-term outcome regarding HRQoL as the presented results are comparable to the large QOLIBRI validation study, that included 20 years younger patients on average [38]. Future long-term outcome studies with larger sample sizes should better stratify for TBI severity subgroups beyond GCS as targeted by the large TRACK-TBI and CENTER-TBI studies. The acute TBI treatment in certified hospitals of the Southern Upper-Bavaria Trauma Network and the subsequent neurorehabilitation in one neurorehabilitation center—using standardized rehabilitation protocols—most probably represent the highest standard of medical care, and therefore results are at least generalizable within industrialized countries.

## Conclusions

The initial TBI severity is a slight contributor to—but not a strong predictor of—HRQoL among chronic TBI patients. The majority of chronic TBI patients reported good HRQoL, comparable to the healthy populations. However, one third of patients suffer from unsatisfactory outcome associated with insufficient autonomy, cognition and increased risk of psychiatric sequelae. Thus, our results suggest the need for further research on early clinical algorithms able to identify patients at risk for unfavorable HRQoL outcome with the risk for psychiatric disorders.

## Supplementary information

**Additional file1: Figure S1.** Cross-sectional study design.

**Additional file 2: Figure S2.** Multivariate regression model to analyze the effect of TBI severity on QOLIBRI total score (model 1). (A) TBI etiology (*p* = 0.83), (B) age at TBI (indirectly calculated), (C) age at survey (*p* = 0.06), and (D) sex distribution (*p* = 0.91) did not correlate with HRQoL. Table S1 shows results in detail.

**Additional file 3: Figure S3.** Multivariate regression model to analyze the effect of TBI severity on subcategories of the QOLIBRI (model 2). Patients reported having good HRQoL in all six subscales. Table S2 shows results in detail.

**Additional file 4: Table S1.** Multivariate regression model to analyze the effect of TBI severity on QOLIBRI total score (model 1). A multivariate regression model (model 1) was established to assess potential contributors to the QOLIBRI total score. HRQoL was modeled as a dependent variable and as a function of the potential contributing factors (i.e., independent variables) listed. The model shows that increasing the GCS by 1-point results in a 1.59-point increase in the QOLIBRI total score. The effect size of model 1 was R^2^ = 0.09, with an adjusted R^2^ = 0.02, indicating a weak correlation. **Table S2.** Multivariate regression model to analyze the effect of TBI severity on subcategories of the QOLIBRI (model 2). The effect of TBI severity (measured using the initial Glasgow Coma Scale score) on HRQoL was calculated for all subscales of the QOLIBRI score (i.e., satisfaction, restrictions, and the six subscales used to determine satisfaction and restrictions). For each component, a model was calculated with each component as a dependent variable and the independent variable of TBI severity. The *p*-value was then extracted for each model, and the effect size of each component is provided as an adjusted R^2^. **Table S3.** Health-related quality of life following TBI: current results in comparison to the QOLIBRI validation study. Comparison between the results obtained in our study (*n* = 135 patients) and the validations cohort by von Steinbuechel et al. (*n* = 795 patients) showing similar results [38].

## Data Availability

The datasets of the current study are available from the corresponding author on reasonable request. All analyzed data on demographics and HRQoL are included in this published article and its supplementary information files.

## Abbreviations

cTBI: Chronic traumatic Brain injury
DAI: Diffuse axonal injury
DALY: Disability-adjusted life years
GCS: Glasgow Coma Scale
HRQoL: Health-related Quality of Life
ICP: Intracranial pressure
mRS: modified Rankin Scale
QoL: Quality of Life
QOLIBRI: Quality of Life after Brain Injury: a questionnaire regarding disease-specific and health-related quality of life in individuals following traumatic brain injury
SD: Standard deviation
SEM: Standard error of the mean
SF: Short Form health survey
SF-8: Short Form (8) health survey: a short and representative form of the SF-36 instrument
SF-12: Short Form (12) health survey: a short form of the SF-36 instrument
SF-36: Short Form (36) health survey: a 36-item patient-reported questionnaire regarding health status, used in medical outcome studies
TBI: Traumatic brain injury
WHOQOL-100: World Health Organization Quality of Life instrument including 100 questions
WHOQOL-BREF: World Health Organization Quality of Life instrument in its abbreviated short form representing the domains of the WHOQOL-100
