# Supplementary figures and images for: Quality of life up to 10 years after traumatic brain injury: a cross-sectional analysis
Source: Health Qual Life Outcomes. 2020 Jun 4;18:166. doi: 10.1186/s12955-020-01391-3 (PMC7271485; doi:10.1186/s12955-020-01391-3)

Supplementary Figure S1

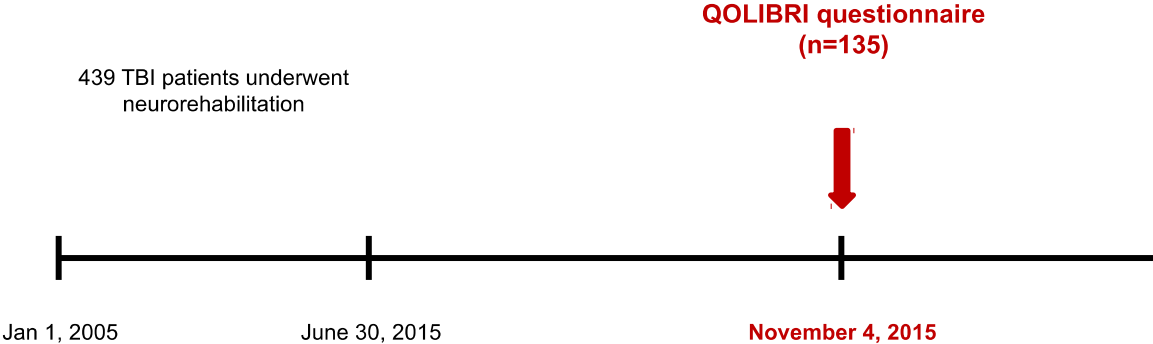

Supplement: Supplementary file 1 — Additional file1: Figure S1. Cross-sectional study design. [file 12955_2020_1391_MOESM1_ESM.pdf]
