# Supplementary material for: Quality of life up to 10 years after traumatic brain injury: a cross-sectional analysis
Source: Health Qual Life Outcomes. 2020 Jun 4;18:166. doi: 10.1186/s12955-020-01391-3 (PMC7271485; doi:10.1186/s12955-020-01391-3)
Supplement: Supplementary file 4 — Additional file 4: Table S1. Multivariate regression model to analyze the effect of TBI severity on QOLIBRI total score (model 1). A multivariate regression model (model 1) was established to assess potential contributors to the QOLIBRI total score. HRQoL was modeled as a dependent variable and as a function of the potential contributing factors (i.e., independent variables) listed. The model shows that increasing the GCS by 1-point results in a 1.59-point increase in the QOLIBRI total score. The effect size of model 1 was R2 = 0.09, with an adjusted R2 = 0.02, indicating a weak correlation. Table S2. Multivariate regression model to analyze the effect of TBI severity on subcategories of the QOLIBRI (model 2). The effect of TBI severity (measured using the initial Glasgow Coma Scale score) on HRQoL was calculated for all subscales of the QOLIBRI score (i.e., satisfaction, restrictions, and the six subscales used to determine satisfaction and restrictions). For each component, a model was calculated with each component as a dependent variable and the independent variable of TBI severity. The p-value was then extracted for each model, and the effect size of each component is provided as an adjusted R2. Table S3. Health-related quality of life following TBI: current results in comparison to the QOLIBRI validation study. Comparison between the results obtained in our study (n = 135 patients) and the validations cohort by von Steinbuechel et al. (n = 795 patients) showing similar results [38]. [file 12955_2020_1391_MOESM4_ESM.pdf]

Supplementary Table S1

	Estimate	SEM	t-value	p-value
(Intercept)	67.13	9.59	7.0	<0.001*
TBI severity (GCS)	1.59	0.76	2.09	0.04*
TBI etiology	-1.24	2.85	-0.21	0.83
Age at survey	-0.28	0.14	-1.93	0.06
Time since TBI	0.18	0.82	0.22	0.83
Sex distribution (female)	-0.64	5.75	-0.11	0.91

Supplementary Table S2

	p-value	adjusted R^2
Level of satisfaction	0.03*	0.10
• Cognition	0.049*	0.05
• Self	0.07	0.08
• Daily life & autonomy	0.03*	0.09
• Social relationships	0.14	0.11
Level of restrictions	0.31	0.08
• Emotion	0.29	0.04
• Physical problems	0.39	0.04

Supplementary Table S3

QOLIBRI item	Results of QOLIBRI cohort n=135	Results from QOLIBRI validation cohort[38] n=795
QOLIBRI total score	65.5 ± 22.6	64.6 ± 18.2
Cognition	62.4 ± 27.2	61.3 ± 21.8
Self	61.1 ± 25.6	60.0 ± 22.0
Autonomy	63.5 ± 31.0	66.4 ± 22.4
Relation	69.3 ± 23.7	63.7 ± 22.6
Emotion	75.0 ± 23.9	71.7 ± 24.7
Physical	66.1 ± 24.1	67.9 ± 23.5
